# Rapid and Efficient Creation of Sweet–Waxy Maize Germplasm via CRISPR/Cas9-Mediated Gene Editing of *Sh2* and *Wx*

**DOI:** 10.3390/cimb48040415

**Published:** 2026-04-17

**Authors:** Xiaolan Yan, Junnan Li, Huijian Liu, Wenfei Jia, Guojun Gao, Yongtian Qin, Longxiang Guan, Xiaxia Duan, Jialu Xu, Pingliang Zhou, Yucai Guo, Xuguang Li, Ling Yang, Hongyu Chen, Weihua Li, Pengshuai Yan, Qingqian Zhou, Zhiyuan Fu, Jihua Tang, Hongqiu Wang

**Affiliations:** 1 State Key Laboratory of High-Efficiency Production of Wheat-Maize Double Cropping, College of Agronomy, Henan Agricultural University, Zhengzhou 450002, China; 2 The Shennong Laboratory, Zhengzhou 450002, China; 3 Hebi Academy of Agricultural Sciences, Hebi 458030, China

**Keywords:** starch synthesis, soluble sugar, amylopectin, molecular breeding, linkage drag, germplasm innovation, Cas9-free

## Abstract

Sweet–waxy maize is a highly valuable specialty maize type with an increasing market demand, but conventional breeding methods for producing sweet–waxy maize are restricted by severe bottlenecks, such as long breeding cycles and linkage drag. This study was conducted to rapidly create sweet–waxy maize germplasm using CRISPR/Cas9 genome-editing technology. We used a CRISPR/Cas9 system to target maize *Sh2* (regulating the super-sweet kernel trait) and *Wx* (controlling the waxy kernel trait), which are two key genes in the starch biosynthesis pathway. Two small-guide RNAs (sgRNAs) designed for each gene were incorporated into CRISPR/Cas9 vectors, which were then introduced into maize via *Agrobacterium*-mediated transformation. We obtained Cas9-free T_3_ homozygous *sh2* and *wx* mutant lines with significant increases in kernel soluble sugar and amylopectin contents, respectively, but no adverse changes to major agronomic traits. Using these Cas9-free lines, we developed a new type of sweet–waxy maize germplasm, in which waxy and sweet kernels on the same ear segregated at a 3:1 ratio. Our results indicate that CRISPR/Cas9-mediated editing of *Sh2* and *Wx* can efficiently generate sweet–waxy maize germplasm with no detectable linkage drag. The study methods would be useful for optimizing the molecular breeding of novel and innovative maize germplasm.

## 1. Introduction

Maize is a highly versatile crop, with a continuously growing market demand for its starch-rich kernels that serve as a critical source of food, feed, and industrial raw materials [1]. Considering the recent increase in the market demand for specialty maize, exploiting fresh maize germplasm resources and breeding texture-specific specialty fresh maize lines have become new priorities for maize breeders and researchers [2]. Sweet maize and waxy maize, which are two typical types of fresh maize, are the result of mutations in different genes associated with the starch biosynthetic pathway. These mutations lead to the excessive accumulation of soluble sugars or amylopectin in kernels, making them potentially useful for maize breeding and agricultural production. Sweet–waxy maize is a specialty type that produces both sweet and waxy kernels on the same ear. On the basis of its unique taste and considerable abundance of nutrients, sweet–waxy maize may have substantial commercial value.

Previous studies elucidated the biochemical mechanisms and genetic characteristics underlying sweet and waxy traits [3,4,5]. Sweet maize varieties are endosperm mutants, with their kernel sweetness determined by sugar and starch contents in the endosperm. In terms of the genetic improvement of sweet maize, identifying mutants with mutations that promote the accumulation of sugar in the endosperm is a major objective. Research conducted to date has confirmed that maize endosperm sweetness is controlled by several recessive genes, including *su1*, *su2*, *sh1*, *sh2*, *bt1*, and *bt2* [6,7,8,9,10,11,12]. By exploiting these genes, breeders have developed standard, super-sweet, and sugar-enhanced sweet maize varieties, all of which have been approved for commercial use [13]. Among these genes, *su1* (controls the sweet kernel trait) and *sh2* (regulates the super-sweet kernel trait) are the most widely used recessive genes for breeding sweet maize. The mutation in *su1*, which encodes a starch debranching enzyme, results in an increase in the endosperm reducing sugar content, with mature kernels that appear shrunken and translucent [6,14]. *Sh2* encodes the large subunit of ADP-glucose pyrophosphorylase (AGPase), which catalyzes the synthesis of ADP-glucose (ADP-Glc) and pyrophosphate from glucose-1-phosphate and adenosine triphosphate, with ADP-Glc serving as the primary substrate for starch synthesis [9]. Decreased AGPase activity due to a mutated *Sh2* will impede starch synthesis and lead to the accumulation of soluble sugars in kernels, ultimately resulting in super-sweet mature kernels that are severely shrunken [15]. Waxy maize, which is also known as glutinous maize, is characterized by mature kernels that contain nearly 100% amylopectin in the endosperm. Furthermore, the endosperm has an opaque and dull waxy appearance, and the kernels have a glutinous texture [16]. The waxy kernel trait is controlled by a recessive mutation in *Wx*, which encodes the enzyme (granule-bound starch synthase I; GBSS I) responsible for elongating the linear chain of glucose polymers in amylose [17,18]. Deleting *Wx* or decreasing GBSS I activity leads to a decrease in the endosperm amylose content, but amylopectin is still synthesized in large quantities. This alters starch gelatinization and swelling properties, resulting in kernels with a waxy texture.

Commercially cultivated sweet and waxy maize hybrids were conventionally developed by introgressing mutant alleles into elite inbred lines via recurrent backcrossing. Typically, six to seven generations of backcrossing with the recurrent parent combined with self-pollination are required to generate genetically stable inbred lines [19]. Recently, genome-editing technology based on Clustered Regularly Interspaced Short Palindromic Repeats (CRISPR) and CRISPR-associated proteins (Cas) has been widely applied, ushering in a new crop breeding era [20,21]. This technology has numerous advantages over other technologies commonly used for breeding. More specifically, it can shorten the breeding cycle, create homozygous genetic backgrounds, and decrease the adverse effects of linkage drag on yield, which helps to explain the increase in its use worldwide [22,23]. Earlier research revealed that CRISPR/Cas9 may be used to target morphogenic genes and create deletion alleles in elite maize inbred lines, with the subsequent multi-location yield testing requiring only 3 years, thereby developing new commercial maize varieties more efficiently than traditional introgression breeding-based approaches [19].

To meet the urgent demand for specialty maize varieties, such as sweet–waxy varieties, this study used CRISPR/Cas9 gene-editing technology to introduce targeted mutations in maize *Wx* and *Sh2* genes. By screening recessive homozygous genotypes and applying combinatorial hybridization techniques, new germplasm materials with sweet and waxy kernels were created. The objective of this study was to apply gene-editing technology to rapidly and efficiently develop new sweet–waxy maize germplasm, while avoiding the adverse effects of linkage drag. The study findings may be relevant to promoting the rapid creation of specialty fresh maize lines with two optimized traits via genome editing. The technical system described herein represents a feasible and efficient breeding model, while the new germplasm resources developed in this study are potentially valuable materials for breeding high-quality sweet–waxy maize varieties, with implications for the fresh maize industry as well as breeding programs.

## 2. Materials and Methods

### 2.1. Plant Materials and Growth Conditions

Maize inbred lines B104 and C01 were used as recipient materials for CRISPR/Cas9 vectors targeting *Wx* and *Sh2*, respectively. These materials were grown in the experimental field of Henan Agricultural University, Zhengzhou, Henan Province, China. Growth conditions were set as previously described [24].

### 2.2. Construction and Transformation of CRISPR/Cas9 Expression Vectors

The CRISPR/Cas9 system was used to target *Sh2* and *Wx* as previously described [25]. Briefly, target sites were screened using CRISPOR (http://crispor.tefor.net/, accessed on 25 May 2021), whereas off-target effects were evaluated using Cas-Offinder (http://www.rgenome.net/cas-offinder/, accessed on 25 May 2021). Fragments containing target sites were amplified by PCR and subsequently cloned into the pBUE411 vector at the *BsaI* restriction site using DNA ligase. Target site sequences are provided in Table S1. A pBUE411 vector carrying two sgRNA sequences was inserted into *Agrobacterium tumefaciens* strain EHA105 cells for the subsequent *Agrobacterium*-mediated transformation of maize plants according to a published method [26].

### 2.3. Molecular Characterization of Transgenic Plants

Genomic DNA was extracted from the leaves of transgenic plants using cetyltrimethylammonium bromide [27]. Genomic sequences containing target sites were amplified by PCR using target-specific primers (Table S1), extracted genomic DNA as the template, and TransStart^®^ FastPfu DNA Polymerase (TransGen, Beijing, China). PCR products were then sequenced using the Sanger method (Qingke, Zhengzhou, China) for sequencing. The obtained sequences were aligned to the maize B73 inbred line reference genome to identify sequence variations in gene-edited plants.

### 2.4. Investigation of the Agronomic Traits of Cas9-Free T_3_ Homozygous Lines

Cas9-free homozygous *Sh2*- and *Wx*-edited lines were obtained via continuous self-pollination up to the T_3_ generation. Several agronomic traits were examined at the kernel milky stage, including plant height, ear height, main tassel length, tassel branch number, upper leaf length and width, ear leaf length and width, and lower leaf length and width. For each line, more than 30 individual plants were analyzed.

### 2.5. Measurement of Kernel Soluble Sugar Contents

Kernel soluble sugar contents were determined on the basis of an anthrone colorimetric assay, which was completed using a Plant Soluble Sugar Content Assay Kit (Solarbio, Beijing, China). Briefly, a pool of 10 kernels from the same ear was ground to a fine powder, after which 0.2 g of ground material was added to a test tube. After adding distilled water, the mixture was incubated in a boiling water bath (100 °C) for 10 min. The mixture was centrifuged (8000× *g* for 10 min at 25 °C) and then the supernatant was collected for the subsequent assay. The reaction system was set, and absorbance at 620 nm was measured as described by the manufacturer’s instructions. Three ears were used as three biological replicates.

### 2.6. Measurement of Kernel Amylopectin Contents

Kernel amylopectin contents were measured using an Amylopectin Content Assay Kit (Solarbio, Beijing, China). Mature kernels from the same ear were ground to a fine powder, after which 0.01 g of ground material was used to determine amylopectin contents according to the manufacturer’s instructions. Three ears were used as three biological replicates.

### 2.7. Iodine–Potassium Iodide Staining of the Kernel Endosperm

Kernel endosperm was visualized via iodine–potassium iodide staining as previously described [19]. Specifically, longitudinal kernel sections were prepared and then a drop of iodine–potassium iodide solution was applied to the endosperm to ensure complete staining. After a 5-min incubation, samples were examined and photographed.

### 2.8. Statistical Analysis

All assays were conducted using three biological replicates, each with three technical replicates. Data are presented as the mean ± standard deviation. Differences among multiple groups were determined by a one-way analysis of variance, which was followed by Tukey’s multiple comparison test for pairwise mean separation. The threshold for significance was set at *p* < 0.05. In the figures presented herein, different lowercase letters indicate significant differences.

## 3. Results

### 3.1. Cloning of gRNA Target Sites and Generation of Sh2- and Wx-Edited Maize Lines

**Figure 1:**
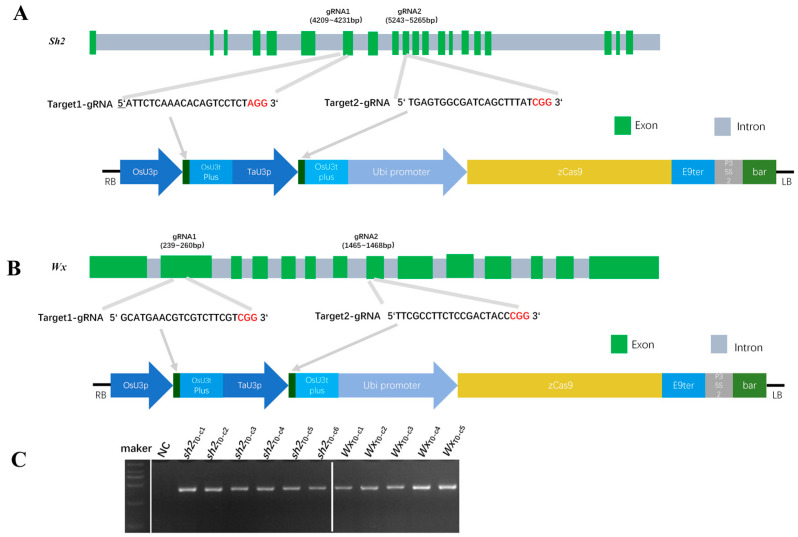
CRISPR/Cas9-mediated targeted mutagenesis of *Sh2* and *Wx* in maize. (**A**,**B**) Construction of CRISPR/Cas9 vectors targeting *Sh2* and *Wx*. *Sh2* (*Zm00001d044129* on chromosome 3) comprises 20 exons and 19 introns, whereas *Wx* (*Zm00001d045462* on chromosome 9) consists of 14 exons and 13 introns. Protospacer adjacent motifs (PAMs) are outlined in red. (**C**) PCR-based screening of *Sh2*- and *Wx*-edited T_0_ transgenic plants using vector-specific primers. NC, negative control.

To create sweet–waxy maize germplasm, we constructed CRISPR/Cas9 gene-editing vectors targeting *Sh2* and *Wx*. In maize, *Sh2* regulates the kernel endosperm soluble sugar content, whereas *Wx* is responsible for the waxy texture of maize kernels. The *Sh2* and *Wx* DNA sequences were deposited at Maize Genetics and Genomics Database (Ames, IA, USA), with accession numbers *Zm00001d044129* and *Zm00001d045462*, respectively. CRISPOR was used to select two gRNA target sequences in exons 6 and 8 of *Sh2* (Figure 1A). These sequences were cloned into the pBUE411 CRISPR/Cas9 expression vector for the *Agrobacterium*-mediated transformation of the C01 maize inbred line. CRISPOR was also used to select two gRNA target sequences in exons 2 and 8 of *Wx* (Figure 1B). These sequences were inserted into the pBUE411 vector for the transformation of the B104 maize inbred line. Transformants were selected using step-wise increases in the bialaphos concentration. A total of 11 T_0_ transformants were confirmed by PCR using vector-specific primers (Table S1), of which six were *Sh2*-edited lines and five were *Wx*-edited lines (Figure 1C).

### 3.2. Characterization of Gene-Edited Sites in T_1_ Lines

**Figure 2:**
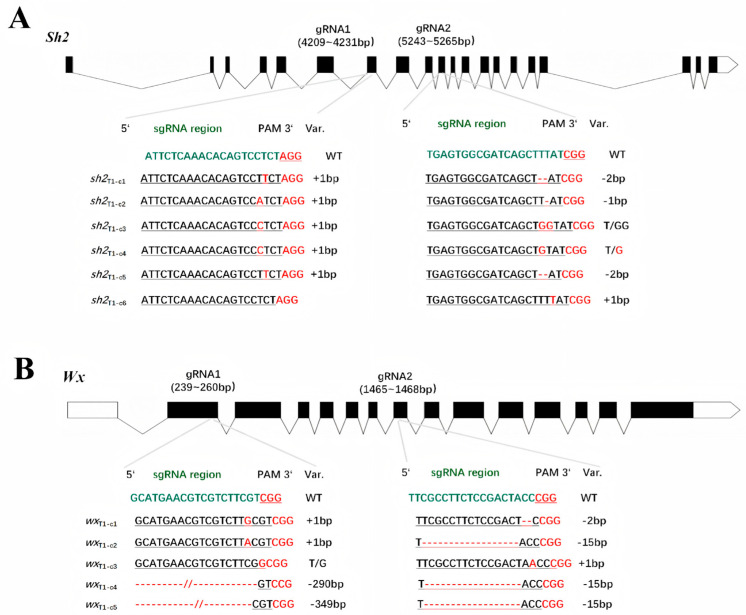
Analysis of target site sequences in T_1_ homozygous gene-edited lines. (**A**) Alignment of mutated sequences in the engineered *sh2* alleles in the C01 genetic background, with the wild-type (WT) sequence as the reference. (**B**) Alignment of mutated sequences in *wx* alleles in the B104 genetic background, with the WT sequence as the reference.

Initially, confirmed T_0_ transformants were self-pollinated to generate T_1_ homozygous gene-edited lines (*Sh2*_T1_ and *Wx*_T1_), from which genomic DNA was isolated. The two *Sh2* target sites were amplified by PCR using gene-specific primers (Table S1) and then sequenced. Sequence analyses revealed that *Sh2* in six T_1_-*Sh2* lines (*sh2*_T1-c1_ to *sh2*_T1-c6_) had four and six distinct mutations at target sites 1 and 2, respectively (Figure 2A). Specifically, *sh2*_T1-c1_ harbored a 1-base pair (bp) insertion (T) at target site 1 and a 2-bp deletion at target site 2. *sh2*_T1-c2_ contained a 1-bp insertion (A) at target site 1 and a 1-bp deletion at target site 2. *sh2*_T1-c3_ had a 1-bp insertion (C) at target site 1 and a T-to-GG substitution at target site 2. *sh2*_T1-c4_ had a 1-bp insertion (C) at target site 1 and a T-to-G point mutation at target site 2. *sh2*_T1-c5_ contained a 1-bp insertion (T) at target site 1 and a 2-bp deletion at target site 2. In *sh2*_T1-c6_, target site 1 was not mutated, but a 1-bp insertion (T) was detected at target site 2.

Sequence analyses of five *Wx*_T1_ lines (*Wx*_T1-c1_ to *Wx*_T1-c5_) revealed five and three distinct mutations at target sites 1 and 2, respectively (Figure 2B). Specifically, *Wx*_T1-c1_ harbored a 1-bp insertion (G) at target site 1 and a 2-bp deletion (AC) at target site 2. *Wx*_T1-c2_ carried a 1-bp insertion (A) at target site 1 and a 15-bp deletion at target site 2. *Wx*_T1-c3_ contained a T-to-G point mutation at target site 1 and a 1-bp insertion (A) at target site 2. *Wx*_T1-c4_ had a 290-bp deletion at target site 1 and a 15-bp deletion at target site 2. Two deletions were detected in *Wx*_T1-c5_ (349 bp at target site 1 and 15 bp at target site 2). Consistent with previous studies, all mutated sites were 3 bp upstream of the NGG PAM at the target sites.

### 3.3. Phenotypic Analysis of Cas9-Free T_3_ Homozygous Sh2-Edited Mutant Lines

**Figure 3:**
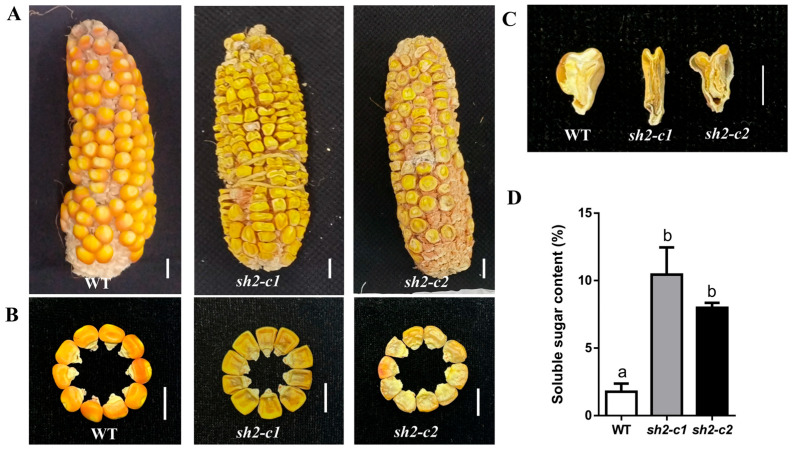
Phenotypic evaluation of Cas9-free T_3_ homozygous *sh2* mutant lines. (**A**) Mature ears of WT and Cas9-free T_3_ homozygous *Sh2*-edited lines (*sh2-c1* and *sh2-c2*); bar = 1 cm. (**B**) Kernel phenotypes of WT, *sh2-c1*, and *sh2-c2* lines; bar = 1 cm. (**C**) Longitudinal sections of kernels from WT, *sh2-c1*, and *sh2-c2* lines; bar = 0.5 cm. (**D**) Soluble sugar contents in WT, *sh2-c1*, and *sh2-c2* kernels. Different lowercase letters in the bar graph indicate significant differences (*p* < 0.05).

Following two consecutive generations of self-pollination and analysis, two Cas9-free T_3_ homozygous *Sh2*-edited mutant lines (*sh2-c1* and *sh2-c2*) were selected for phenotypic characterization. In both lines, mutations in *Sh2* resulted in the production of truncated proteins comprising 167 amino acids (Figure S1). Additionally, in contrast to mature wild-type (WT) kernels, the mature mutant kernels were shrunken and translucent, with a depressed apex (Figure 3A,B). To further characterize internal structural changes, mature kernel longitudinal sections were examined, which revealed that the insufficient accumulation of starch in the endosperm of *sh2-c1* and *sh2-c2* kernels resulted in gaps (Figure 3C). This kernel phenotype was attributable to the loss of AGPase activity, which negatively impacted the conversion of soluble sugars to starch. Soluble sugar contents in *sh2-c1* and *sh2-c2* mutant kernels were measured at 20 days after pollination (DAP). The results indicated that soluble sugar contents were significantly higher in mutant kernels than in WT kernels (Figure 3D). Additionally, other agronomic traits (e.g., plant height, ear height, main tassel length, number of tassel branches, flag leaf length, flag leaf width, ear leaf length, ear leaf width, lower leaf length, and lower leaf width) did not differ significantly between the two mutant lines (*sh2-c1* and *sh2-c2*) and the WT control (Figure S2). Considered together, these results indicate that *sh2* mutations primarily affected the maize kernel sugar content, with no significant effects on other agronomic traits.

### 3.4. Phenotypic Evaluation of Cas9-Free T_3_ Homozygous Wx-Edited Mutant Lines

**Figure 4:**
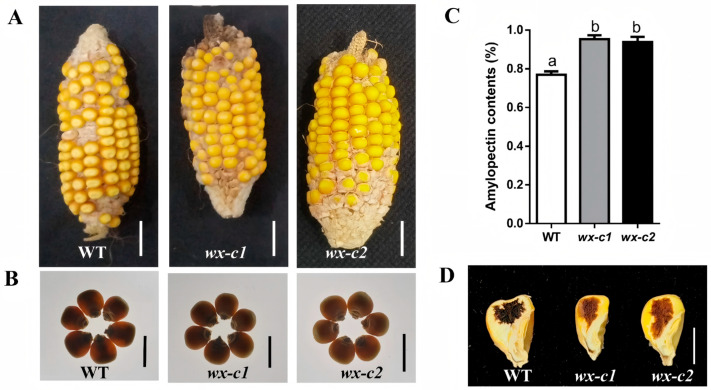
Phenotypic evaluation of Cas9-free T_3_ homozygous *wx* mutant lines. (**A**) Mature ears of WT and Cas9-free T_3_ homozygous *Wx*-edited lines (*wx-c1* and *wx-c2*); bar = 2 cm. (**B**) WT, *wx-c1*, and *wx-c2* kernels viewed on a light box; bar = 1 cm. (**C**) Amylopectin contents in WT, *wx-c1*, and *wx-c2* kernels. Different lowercase letters in the bar graph indicate significant differences (*p* < 0.05). (**D**) WT, *wx-c1*, and *wx-c2* kernels stained with iodine–potassium iodide solution; bar = 0.5 cm.

To obtain stable Cas9-free homozygous *Wx*-edited mutant materials, two consecutive generations of self-pollinated *Wx*_T1_ lines were screened. Two Cas9-free T_3_ homozygous *Wx*-edited mutant lines (*wx*-*c1* and *wx*-*c2*) were selected for phenotypic and biochemical analyses. In both mutant lines, mutations in *Wx* resulted in the production of truncated proteins consisting of 163 amino acids (Figure S3). Phenotypic analyses of mature ears revealed that *wx-c1* and *wx*-*c2* kernels had a convex apex, but a distinct dull appearance unlike that of WT kernels (Figure 4A). To further examine the mutant kernel phenotype, mature kernels were placed on a light box to analyze translucency. Light transmittance was significantly lower for *wx*-*c1* and *wx*-*c2* kernels than for WT kernels (Figure 4B), likely because of the waxy endosperm composition of the kernels produced by these *Wx*-edited lines. Amylopectin contents of *wx*-*c1* and *wx*-*c2* kernels were determined at 20 DAP. According to the results, the kernels of these *Wx*-edited lines contained significantly more amylopectin than the WT kernels (Figure 4C). This increase in the amylopectin content of the mutant kernels was attributed to the inactivation of GBSS I, which resulted in impaired amylose synthesis. Additionally, *wx*-*c1* and *wx*-*c2* kernels were stained with iodine–potassium iodide solution to elucidate starch characteristics. Consistent with the fact that amylopectin typically turns brown after being stained with a potassium iodide solution, *wx*-*c1* and *wx*-*c2* kernel endosperm had a light tan color after iodine–potassium iodide staining. By contrast, the endosperm of WT kernels was dark blue (Figure 4D). These observations were in accordance with the waxy kernel phenotype of *Wx*-edited lines. Furthermore, similar to the two *Sh2*-edited lines, the *wx-c1* and *wx*-*c2* mutant lines did not differ significantly from the WT control in terms of 10 other agronomic traits (Figure S4). Collectively, these findings suggest that mutating *Wx* can significantly increase the maize kernel endosperm amylopectin content without significantly altering key agronomic traits.

### 3.5. Creation of New Sweet–Waxy Maize Germplasm

**Figure 5:**
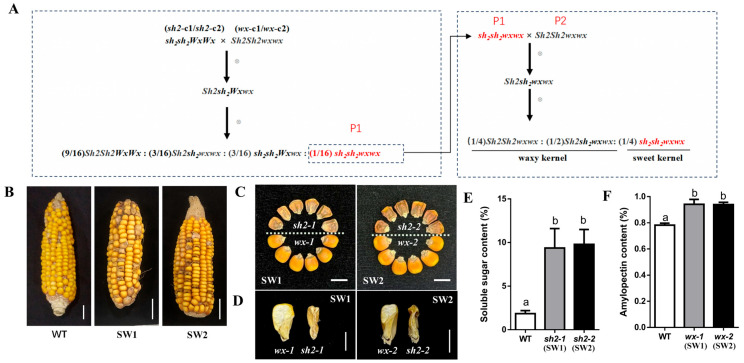
Creation of sweet–waxy maize germplasm. (**A**) Genetic basis for the creation of sweet–waxy (SW) maize. Two crosses (*sh2-c1* × *wx-c1* and *sh2-c2* × *wx-c2*) were completed to generate hybrids with the *Sh2sh2Wxwx* genotype. (**B**) Mature ears of WT and SW (SW1 and SW2) maize; bar = 2 cm. (**C**) Phenotypes of sweet kernels (*sh2-1* and *sh2-2*) and waxy kernels (*wx-1* and *wx-2*) from SW1 and SW2 ears; bar = 1 cm. (**D**) Longitudinal sections of waxy (*wx-1* and *wx-2*) and sweet (*sh2-1* and *sh2-2*) kernels from SW1 and SW2 ears; bar = 0.5 cm. (**E**) Soluble sugar contents of sweet kernels from SW1 and SW2 ears. (**F**) Amylopectin contents of waxy kernels from SW1 and SW2 ears. Data are presented as the mean ± standard deviation of three independent samples per genotype. Different lowercase letters in the bar graphs indicate significant differences (*p* < 0.05).

Cas9-free homozygous *Sh2*- and *Wx*-edited lines were highly valuable for the breeding of sweet–waxy composite maize germplasm. Accordingly, *sh2*-*c1*/*sh2*-*c2* mutant lines were crossed with *wx*-*c1*/*wx*-*c2* mutant lines to generate F_1_ hybrids with the *SH2sh2WXwx* genotype. After self-pollinating the F_1_ hybrids, progenies with the *sh2sh2wxwx* genotype were isolated and used as P1 parental lines for generating sweet–waxy maize germplasm, with *wx-c1*/*wx-c2* lines with the *SH2SH2wxwx* genotype serving as P2 parents. Crossing P1 and P_2_ produced F_1_ hybrids with the *SH2sh2wxwx* genotype. Subsequent self-pollination of these F_1_ hybrids generated sweet–waxy composite ears (SW1 and SW2), with waxy and sweet kernels segregated at a 3:1 ratio (Figure 5A,B, Table S2).

Phenotypic analyses of kernels on mature SW1 and SW2 ears indicated that in contrast to waxy kernels, sweet kernels were shrunken and relatively small (Figure 5C). Consistent with this observation, longitudinal sectioning and the subsequent comparison with waxy kernels revealed the insufficient starch filling and severe endosperm shrinkage in sweet kernels (Figure 5D). Sweet and waxy kernels were collected from SW ears at 20 DAP for analyses of soluble sugar and amylopectin contents, respectively. Notably, soluble sugar contents were significantly higher in sweet kernels than in WT kernels (Figure 5E). Similarly, amylopectin contents were significantly higher in waxy kernels than in WT kernels (Figure 5F). These results confirmed that a novel sweet–waxy maize germplasm was developed.

## 4. Discussion

Sweet maize and waxy maize are important specialty maize types. There has been a steady increase in the market demand for sweet–waxy maize varieties because they combine the sugary flavor of sweet maize with the soft and glutinous texture of waxy maize. However, most currently cultivated varieties are conventional maize germplasm derived from a few natural mutants that are decades old [28]. The utility of traditional breeding methods is limited by technical bottlenecks, such as long breeding cycles, severe linkage drag, inefficient target trait segregation, and interference from epistatic effects between genes [28,29,30]. In the current study, we used a CRISPR/Cas9 genome-editing system to target maize genes *Sh2* and *Wx* for editing, which resulted in the generation of homozygous mutants free of Cas9. Mature kernels of the *sh2* mutant had a typical shrunken phenotype, with insufficient endosperm starch contents and significantly increased soluble sugar contents at 20 DAP (Figure 3A–D). These kernel traits were in accordance with the kernel characteristics of previously reported *sh2* mutants [15,31]. Notably, homozygous *sh2* mutants did not differ significantly from the WT control in terms of 10 major agronomic traits, including plant height, ear height, and leaf traits (Figure S2). Accordingly, the targeted editing of *Sh2* specifically modulated sugar metabolism in maize kernels, but there was no significant pleiotropy or linkage drag. This is in contrast to the frequent association between recessive mutant alleles and undesirable agronomic traits during traditional backcross breeding. In addition, the homozygous *wx* mutant generated using CRISPR/Cas9 gene-editing technology produced ears with waxy kernels, with no significant changes in the other analyzed agronomic traits (Figure S4). These findings reflect the specificity of the sgRNAs designed in this study and the precision of the targeted gene editing. Hence, the other examined agronomic traits were not adversely altered by off-target effects. The generated germplasm may be applicable for breeding new waxy maize varieties.

CRISPR/Cas9-mediated genome editing involves the introduction of double-strand breaks at the targeted genomic regions, which are subsequently repaired through non-homologous end-joining or homology-directed repair pathways [32]. In plant somatic cells, non-homologous end-joining is the major repair pathway, but it often results in insertions, deletions, and/or substitutions that lead to gene knockout [33]. The CRISPR/Cas9 system uses sgRNAs to target the genome, but the targeting efficiency may vary among sgRNAs. Therefore, using two sgRNAs may increase target gene knockout efficiency. The results of this study showed that among the six *sh2* T_1_ lines, the target site 2 was edited in all lines, with mutation types including insertion, deletion, and substitution. By contrast, target site 1 was edited in only five lines, all of which exhibited single-base insertions (Figure 2A). In the five T_1_ *wx* mutant lines, the deleted fragment at target site 1 was up to 349 bp long, whereas the longest deletion at target site 2 was 5 bp (Figure 2B). These differences in gene-editing efficiency and editing types are likely related to differences in sgRNA design and target site accessibility, which can affect the ability of the CRISPR/Cas9 system to precisely and efficiently edit genomic targets. Using paired sgRNAs and a CRISPR/Cas9 system to target dual sites may lead to the deletion of genomic fragments between the two target sites. In plants, short genomic deletions of approximately 100 bp have been frequently reported [34,35]. Notably, such inter-target genomic deletions were undetectable in the *sh2* and *wx* mutant lines examined in this study, possibly because of the relatively large distance between the two designed target sites (approximately 1000 and 1800 bp, respectively). These results suggest that the efficiency of genomic deletion varies considerably depending on the fragment length between the two target sites, with short deletions (approximately 100 bp) more easily achieved than large deletions, which is consistent with the findings of previous studies [34,35,36].

In summary, in this study, maize *Sh2* and *Wx* genes were precisely edited using CRISPR/Cas9, with the Cas9-free homozygous sweet and waxy mutants exhibiting stable agronomic traits. We also established an efficient strategy for developing novel sweet–waxy maize germplasm that can produce ears containing both sweet and waxy kernels. In future studies, integrating our approach with haploid technology may overcome the technical bottlenecks of conventional sweet–waxy maize breeding methods, thereby enabling *Sh2* and *Wx* to be efficiently and precisely edited regardless of the genetic background. Furthermore, this strategy may be relevant to the genetic improvement of other crops.

## Data Availability

The original contributions presented in this study are included in the article/Supplementary Materials. Further inquiries can be directed to the corresponding authors.

## Supplementary Materials

The following supporting information can be downloaded at: https://www.mdpi.com/article/10.3390/cimb48040415/s1.
