# Supplementary figures and images for: Rapid and Efficient Creation of Sweet–Waxy Maize Germplasm via CRISPR/Cas9-Mediated Gene Editing of Sh2 and Wx
Source: Curr Issues Mol Biol. 2026 Apr 17;48(4):415. doi: 10.3390/cimb48040415 (PMC13115123; doi:10.3390/cimb48040415)

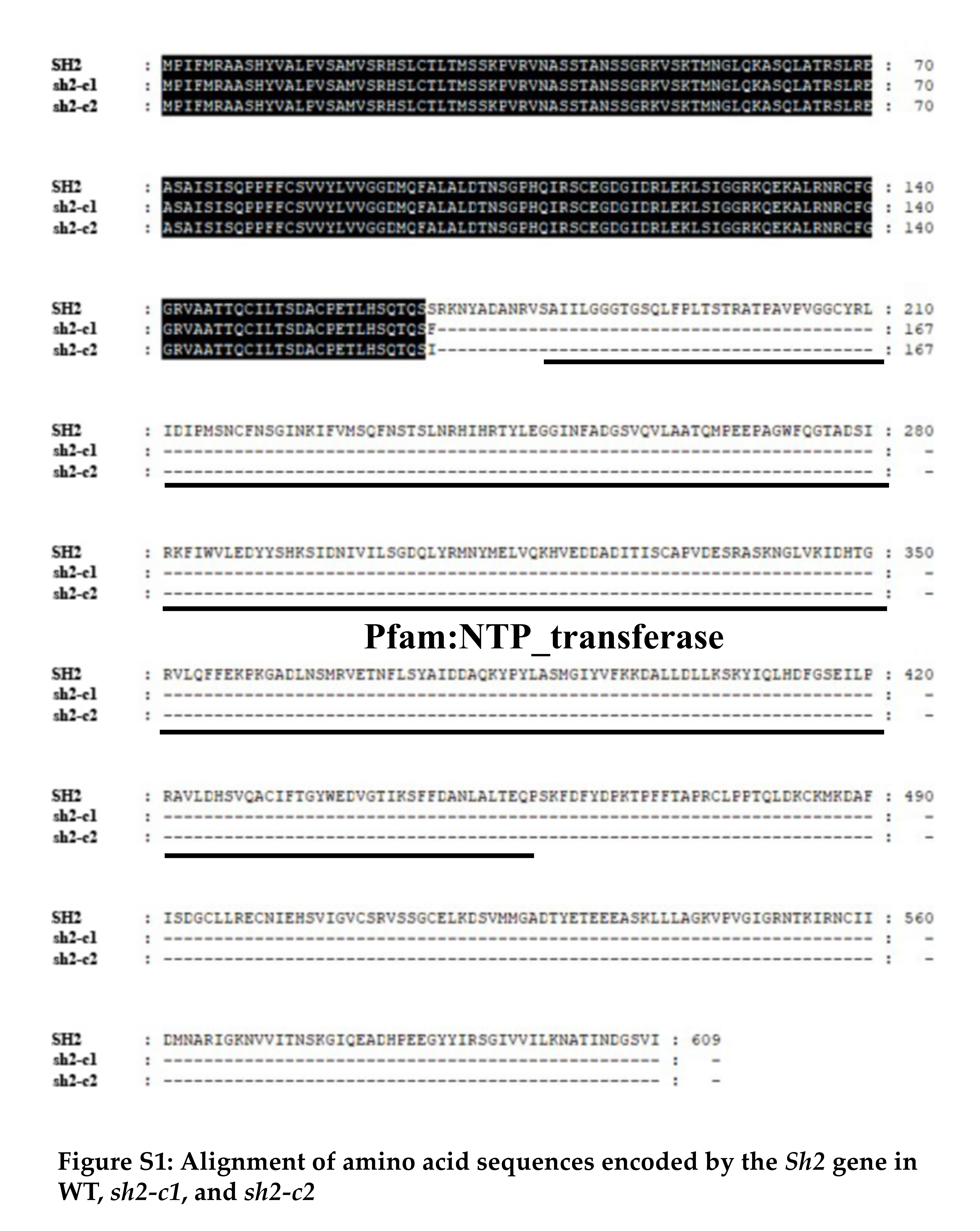

Supplement: Supplementary file 1 [file cimb-48-00415-s001.zip › Supplement Figure S1.png]

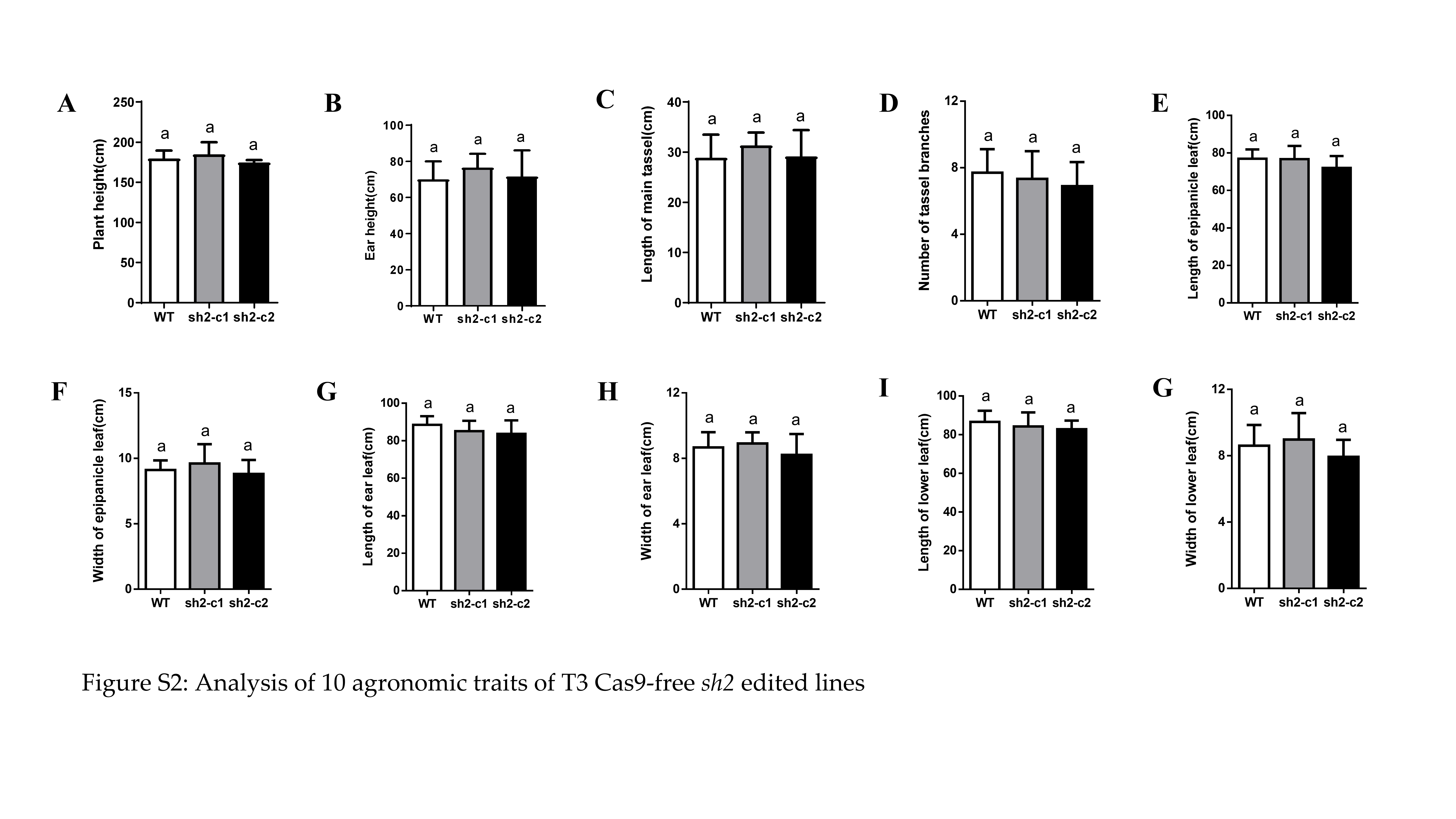

Supplement: Supplementary file 1 [file cimb-48-00415-s001.zip › Supplement Figure S2.png]

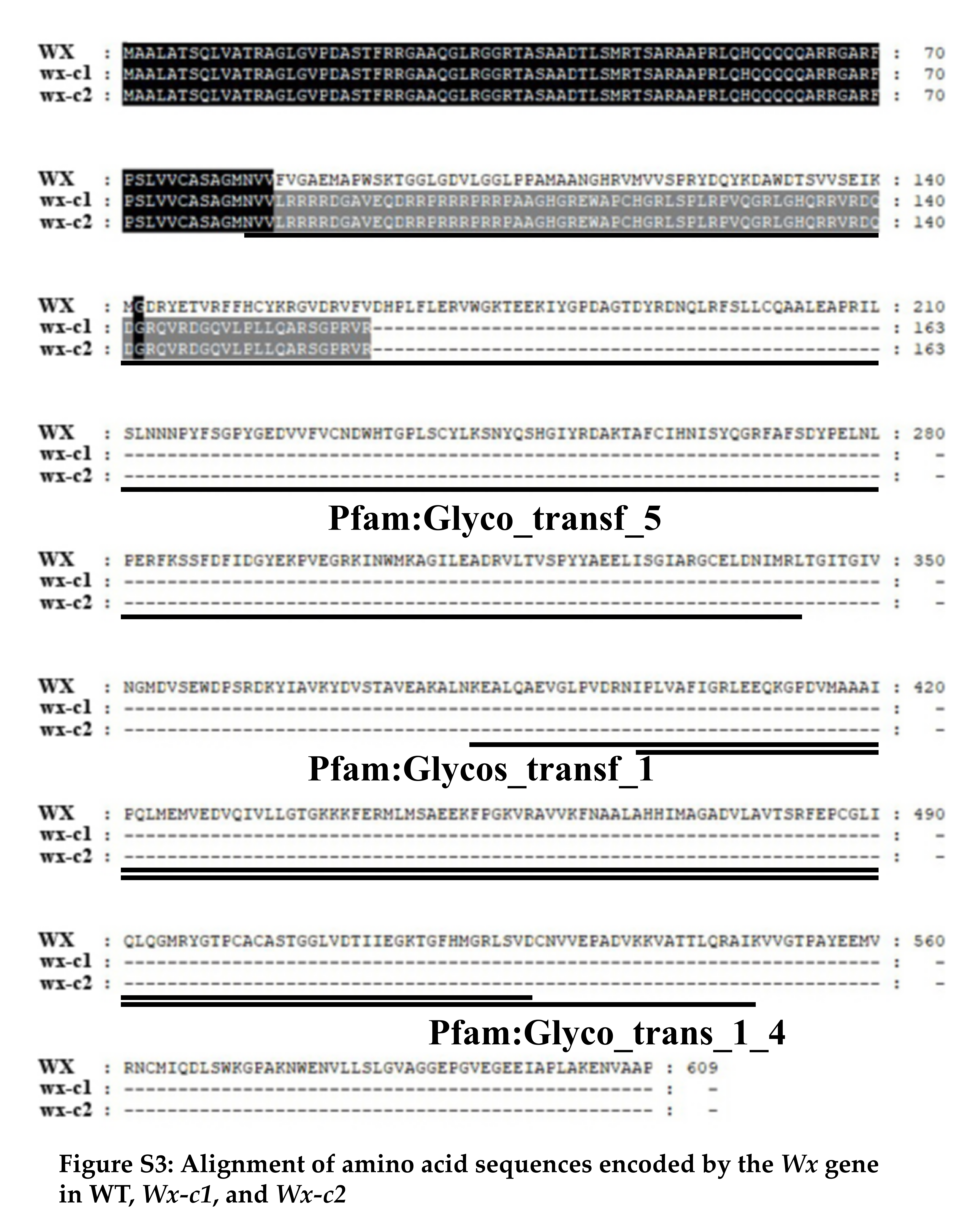

Supplement: Supplementary file 1 [file cimb-48-00415-s001.zip › Supplement Figure S3.png]

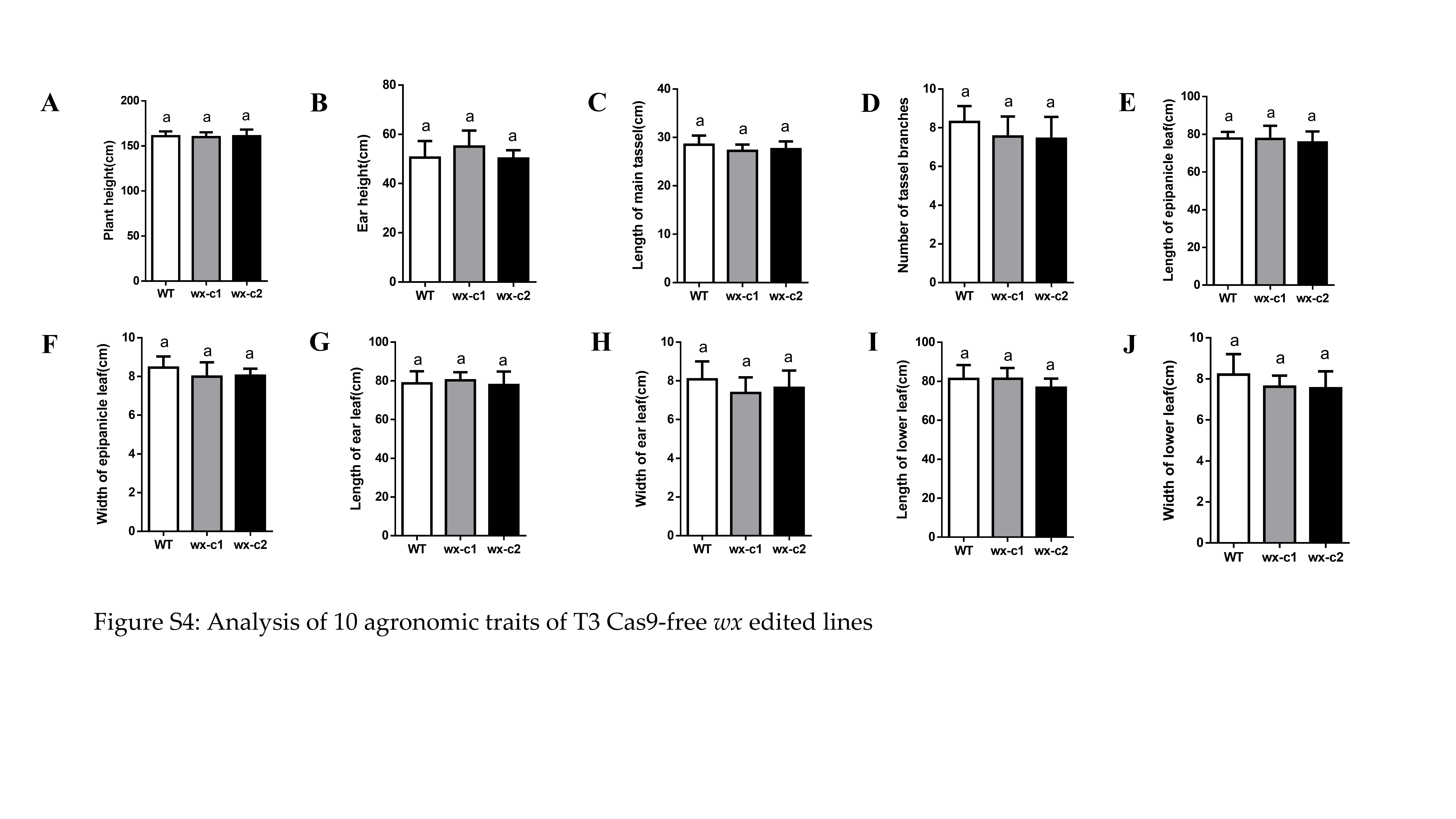

Supplement: Supplementary file 1 [file cimb-48-00415-s001.zip › Supplement Figure S4.png]
